# Access Control for IoT: A Survey of Existing Research, Dynamic Policies and Future Directions

**DOI:** 10.3390/s23041805

**Published:** 2023-02-06

**Authors:** Kaushik Ragothaman, Yong Wang, Bhaskar Rimal, Mark Lawrence

**Affiliations:** 1 College of Business and Information Systems, Dakota State University, Madison, SD 57042, USA; 2 The Beacom College of Computer and Cyber Sciences, Dakota State University, Madison, SD 57042, USA

**Keywords:** Internet of Things, identity and access management, access control, authorization, policies specification

## Abstract

Internet of Things (IoT) provides a wide range of services in domestic and industrial environments. Access control plays a crucial role in granting access rights to users and devices when an IoT device is connected to a network. However, many challenges exist in designing and implementing an ideal access control solution for the IoT due to the characteristics of the IoT including but not limited to the variety of the IoT devices, the resource constraints on the IoT devices, and the heterogeneous nature of the IoT. This paper conducts a comprehensive survey on access control in the IoT, including access control requirements, authorization architecture, access control models, access control policies, access control research challenges, and future directions. It identifies and summarizes key access control requirements in the IoT. The paper further evaluates the existing access control models to fulfill the access control requirements. Access control decisions are governed by access control policies. The existing approaches on dynamic policies’ specification are reviewed. The challenges faced by the existing solutions for policies’ specification are highlighted. Finally, the paper presents the research challenges and future directions of access control in the IoT. Due to the variety of IoT applications, there is no one-size-fits-all solution for access control in the IoT. Despite the challenges encountered in designing and implementing the access control in the IoT, it is desired to have an access control solution to meet all the identified requirements to secure the IoT.

## 1. Introduction

Internet of Things (IoT) provides many conveniences to users in domestic and industrial environments. Statista estimated that 75 billion devices would be connected to the Internet worldwide by 2025 [1]. As more devices are connected to the Internet, many attacks have been reported targeting to IoT devices [2,3,4]. Security and privacy are two major concerns revolving around the IoT. Imagine the world where billions of “things”, e.g., vehicles, buildings, appliances, and mobile devices, are connected to the Internet. The impact of any data breaches and security incidents will be enormous. IoT device manufacturers and service providers are required by regulations to ensure security of their devices, thereby protecting users’ privacy.

Security services such as authentication, access control, confidentiality, integrity, availability, and non-repudiation are essential to secure the IoT. Among all the security services, access control ensures appropriate access to resources across users, devices, applications, and services. Access control is essential in implementing security for any IoT application [5,6]. For example, if a car manufacturer wants to sell smart cars to its customers, the manufacturer must design the vehicle’s system in a way that it constantly collects and processes data from its surrounding environment through sensors embedded in the vehicle. The system might occasionally transmit the collected data to the manufacturer via the Internet. The data may contain the driver’s personal information and sensitive information such as locations. The data that is being sent and shared with the manufacturer should only be accessed by authorized users. Furthermore, appropriate access controls must be placed on the shared data if the manufacturer facilitates remote start functions for vehicle owners.

Access control provides the desired service to protect against the unauthorized use of the accessible resources. Traditional access control models are being adopted or extended for access provisioning and management in the IoT. However, the design and implementation of access control for the IoT are complicated. IoT networks include devices with different hardware and software configurations. Their heterogeneous nature raises a considerable challenge for any access control solution. In addition, IoT devices are resource-constrained devices with limited memory, computation power, and battery [7]. The constrained resources on the IoT devices limit the use of complex algorithms when designing an access control solution. Further, IoT networks have encountered major attacks in recent years on a global scale [2,3,4]. Governments of many countries have already initiated to formulate policies for IoT devices. Hence, an appropriate access control solution is required for any IoT network.

This paper conducts a comprehensive survey of access control in the IoT, including access control requirements, authorization architecture, access control models, access control policies, access control research challenges, and future directions. A few survey articles discussed IoT access control [8,9,10,11,12,13]. However, none of them discuss the issues regarding the IoT dynamic access control policies. To the best of our knowledge, this is the first survey paper reviewing the IoT access control dynamic policies’ issues. The key contributions of the paper are summarized as follows.

- The latest development of the access control in the IoT is provided to understand the recent progress on the access control.
- Access control requirements are discussed to help design and implement access control solutions for effective identity and access management in the IoT.
- Three major access control authorization architectures, namely, policy-based, token-based open authorization, and hybrid user-managed access architectures are discussed, and their essential components are briefly summarized.
- We compare different IoT access control models, including discretionary access control, role-based access control, attribute-based access control, organization-based access control, usage-based access control, capability-based access control, blockchain-based access control, and relationship-based access control to facilitate the adoption of access control solutions.
- Access control policies such as dynamic policies’ specification are thoroughly discussed. The challenges faced by the current solutions are highlighted.
- To guide future research in access control, we summarize the research challenges in access control and also point out future research directions in the IoT.

The remainder of this paper is organized as follows: Section 2 presents an overview of access control in the IoT. Section 3 introduces access control authorization architectures, followed by discussions of access control models and policies’ specification in Section 4 and Section 5, respectively. Section 6 presents the research challenges and future directions in the access control in the IoT, and finally, Section 7 concludes the paper.

## 2. Access Control in the IoT

IoT interconnects computing devices embedded in everyday objects. IoT has been widely adopted in consumer and business environments to bring convenience and facilitate business processes. Due to the large amount of data IoT collects and the sensitivity of the data, IoT security is critical [14].

### 2.1. Access Control

Access control includes access to both information systems and physical facilities [15]. This paper reviews the techniques to enable “the process of granting or denying specific requests to obtain and use information and related information processing services [15]” in the IoT. Access control to specific physical facilities is outside the scope of this paper.

**Figure 1:**
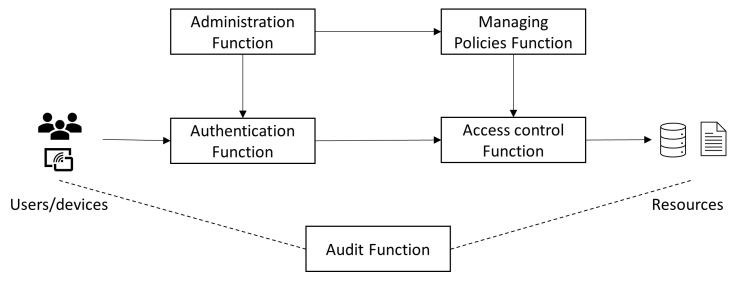
Access Control Process in the IoT.

Access control plays a crucial role in granting access rights to users and devices when IoT devices are connected to a network. An access control process generally includes functions such as authentication function, access control function, audit function, managing policies’ function, and administration function, as shown in Figure 1. The authentication function verifies the identity of a user, a process, or a device. Access control function grants and denies specific requests from a user, a process, or a device to access resources. Access control policies describe high-level requirements that specify how access is managed and who may access information under what circumstances. The access control process also includes an administration function to create, provision, and effectively manage different users, groups, roles, devices, and policies. The audit function provides an independent review and examination of records and activities to assess the adequacy of access control to ensure compliance with established policies and operational procedures. Authorization takes place after authentication is complete. Authorization is also coupled with authorization policies to determine which resources/services are available to a user or a device. Section 3 introduces three common access control authorization architectures in the IoT.

### 2.2. Access Control Requirements

IoT presents many challenges for access control. The work in [9] identifies six requirements that should be satisfied by any IoT application. These requirements include scalability, interoperability, performance, reliability and availability, dynamicity, and usability [9]. The requirements in [9] are not specific to the IoT and may apply to other devices. Based on the work in [9,16], we further refined these requirements based on the characteristics of the IoT, including but not limited to the variety of the IoT devices, the resource constraints on the IoT devices, and the heterogeneous nature of the IoT. The twelve refined requirements, which are more specific to the IoT, are summarized below.

1. Granularity: Granularity is the expressiveness of the policies used to formulate access control rules [17]. The fine-grained nature is the most important characteristic of any solution that is designed to manage access rights. Due to the heterogeneity property of the IoT networks and their dynamic nature, granularity is a major concern while designing access control models [9,18].
2. Policies’ Specification: Policies developed for access control models should be able to handle dynamicity and allow and monitor delegation. An IoT network may contain a large number of devices presented in various forms and locations. Therefore, access control should consider the granularity and the policies’ specification to govern the network effectively [9].
3. Handling Complexity: IoT networks are heterogeneous networks that are characterized by resource-constrained devices, multiple hop links, unreliable communications, and limited physical security. Access control models shall be designed to handle the complex nature of the IoT networks [10].
4. Interoperability: Many device manufacturers provide a variety of IoT devices to customers. There is a high possibility that an IoT network may contain devices from different manufacturers and must function together. Therefore, access control must support this interoperable nature in the IoT [19].
5. Facilitation of Users: IoT devices may be shared and accessed by multiple users. For example, virtual assistants and smart home products can be used by family members and guests at home. Access control must be able to allow users to delegate access to other users instead of handling them all at a single administrative point [20,21].
6. Automation: The complex nature of IoT environments and the number of access decisions to be made at a given time make it difficult to provision or make decisions individually. Hence, the processes of policy generation, decision and evaluation should be automated in the IoT [18,22].
7. Resource Constraints: IoT devices possess low memory and processing power when compared to regular computing machines [7]. Many IoT devices also operate on battery power. These constraints raise challenges in developing access control solutions for the IoT [10,23].
8. Coherence: In the case of multiple administrative points adopted in access control, all the administrative nodes should be coherent when managing and provisioning access control. The variant types of IoT networks create a challenge when ensuring coherence across multiple administrative domains in the IoT [9].
9. Resolving Identities: Access control grants or denies requests from a user, a device, an application, or a service. It assumes that each user, device, application, or service is uniquely identified. IoT devices can be characterized by attributes such as model numbers, serial numbers, IP addresses, physical addresses, locations, etc. In turn, these devices are accessed by other devices and human users when connected to a network. Leveraging a combination of the device attributes to uniquely identify a device in a network poses a challenge during the access control specification and implementation [18].
10. Downtime: Downtime is the amount of time when access control is not available. The dynamic nature of the IoT environments tests the limits of any access control solution. Since access decisions are made frequently, there should be no downtime [8,9,22]. The design of a centralized model or a distributive model decides the downtime. In a centralized model, if the administrative node fails, it causes a single point of failure.
11. Scalability: Scalability is the ability for any access control process to continue to function properly when the number of users and devices changes [24]. Due to the vast number of devices available, access control in the IoT must be extensible to support the number of users and devices, the variety of devices, and the heterogeneous structures in the IoT [8].
12. Security: Access control is an essential process in any information system. The security of the access control process itself is thus important. Software defects such as design flaws and implementation bugs could be exploited by malicious actors [25]. Thorough security analysis should be conducted to ensure access control solutions are resistant to any cyber attack [10,14,23].

### 2.3. Discussion

Policies play an important role in access control. Access control decisions are governed by access control policies which pave the way for other requirements to be satisfied. Specifying effective policies prevents over privilege and data leakage, thereby ensuring the security of a system. IoT networks are heterogeneous and dynamic in nature. Communications may occur among users, devices, applications, and services. Hence, access control policies must be granular in nature. To enforce access control policies, users and devices must be uniquely identified. Access control policies must allow specification in terms of context. The fine-grained and context-aware nature of access control policies helps fulfill the requirements of scalability, complexity, and interoperability. Since multiple policy administration points may be used in a solution, access control must support the distributed nature. Supporting a distributed architecture also brings coherence to policy management and reduces downtime. On the other hand, an access control process must facilitate users by specifying policies that allow them to delegate access rights to others. Smart home platforms such as Amazon [26], Google [27] and SmartThings [28], as well as commercial home automation platforms such as IFTTT [29], Zapier [30] and Power Automate [31] facilitate users to specify rules using natural language statements. IoT applications in development must fulfill the requirement to facilitate the users to delegate permissions. Finally, to satisfy the requirements of granularity and scalability in the IoT, it is efficient only if the entire policy management is automated.

## 3. Access Control Authorization Architecture

Access control is primarily implemented within centralized and distributed architecture categories in the IoT. In a centralized architecture, a single node is used for policy administration and management, i.e., access provisioning and revocation happening from a single entity [10]. One of the limitations in centralized architecture is the single point of failure. In a dynamic environment such as IoT, the entity that administers access control decisions is expected to be available anytime.

Distributed architecture, in contrast, can handle multiple nodes for administration [10]. Although it is easier to facilitate delegation and scalability, a challenge in designing access control solutions for distributed architecture is coherence. A decision or a change made at one node should reflect in all the other managing nodes. Designing an appropriate access control solution depends on the architecture of an IoT network.

There are different types of authorization architectures available. The common types are the policy-based architecture, the token-based Open Authorization (OAuth) architecture, and the hybrid User-Managed Access (UMA) architecture [8]. Other customized architectures are either derived from those three or specific to the proposed applications.

### 3.1. Policy-Based Architecture

A policy-based architecture may include Policy Enforcement Point (PEP) to perform access control, Policy Decision Point (PDP) to offer authorization, Policy Information Point (PIP) as a source of attributes, Policy Administration Point (PAP) to create and administer the policy, and Policy Refinement Point (PRP) to refine policies at runtime. These components are briefly discussed below.

1. Policy Administration Point: The PAP, also known as a policy repository, is where all the policies required to grant or deny permissions are stored. Typically, the policies are stored in a specific format, for example, Extensible Access Control Markup Language (XACML). In addition, the PAP makes the complete access control policies available for the policy decision point to grant or deny permissions [32,33]. In an IoT environment, PAP should be designed so that policies can be added, removed or modified at runtime.
2. Policy Enforcement Point: The PEP acts as an intercept between the PDP and the requesting subjects. It forwards every request made by a subject along with the attribute values related to the subjects, the resources, the actions to be performed, and the environment to the PDP. Once the evaluation is performed at the PDP, the PEP retrieves the decision and forwards it to the subject that made the request. Moreover, based on the decision, the PEP is responsible for enforcing the actions that the subject can perform on a resource (e.g., read, write or both) [32,34,35].
3. Policy Decision Point: The PDP evaluates the requests it receives based on the subject that makes the request, the resource that the subject is requesting to access, and the contextual (attributes) information. The PDP triggers the PIP to provide all the required contextual information, such as attribute values of the requester, the resources, the action that is being requested, and the environmental variables. Based on the information that is received, the PDP evaluates the decision by verifying them against the policies [32,34,35].
4. Policy Information Point: The PIP is responsible for collecting and storing all the contextual information related to the system. In an IoT network, granting or denying permissions based on context is one of the important requirements of access control. Hence, whenever the PDP requires the contextual information and the attribute information, the PIP sends them through the PEP to make an access decision [32,34,35].
5. Policy Refinement Point: The PRP is a component that is responsible for refining policies at runtime and updating the policy repository. The refining process can be triggered for several reasons such as any change in the context of the environment or detection of an abnormal or unauthorized access behavior [33,36]. Various techniques have been adopted in the literature for the policy refinement process [32,33,34,37]. Most of these techniques are based on artificial intelligence. The PRP contributes to automating policies’ specification for access control which is essential in a dynamic environment such as IoT.

**Figure 2:**
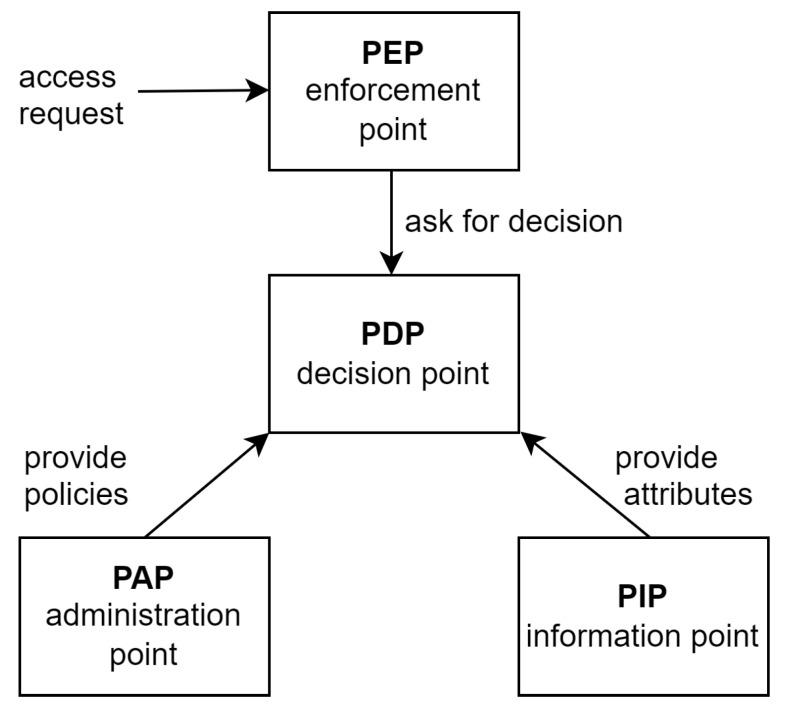
XACML Architecture [8].

XACML demonstrates a policy-based architecture as shown in Figure 2. It includes four essential components including PEP, PDP, PAP, and PIP. XACML is a popular standard that provides fine-grained access control. It is based on the Extensible Markup Language (XML), which is standardized by the OASIS consortium [8].

### 3.2. Token-Based OAuth Architecture

OAuth is an open-source authorization standard that is mainly used to provide access to web applications and services. With OAuth, users can access protected resources to third-party applications without disclosing their login credentials. Major OAuth service providers include Google, Microsoft, and Facebook [8]. These service providers are identity providers which verify the users and provide external applications access to the users’ information stored on the providers’ domains with the users’ consent.

**Figure 3:**
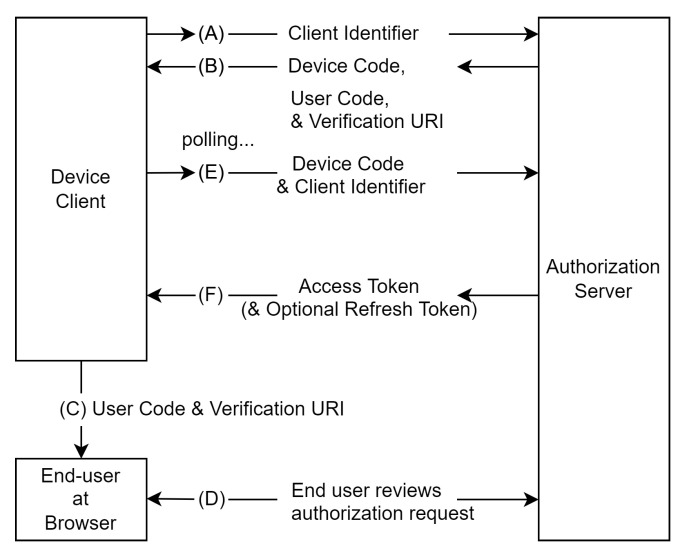
OAuth Device Authorization Flow [38].

The Internet Engineering Task Force (IETF) has extended OAuth 2.0 for devices, and browserless clients under RFC8628 [38]. Figure 3 shows the OAuth device authorization flow. As shown in the figure, the flow is the sequence of steps (A) through (F). The client initially sends an access request along with its client identifier to the authorization server. Following the request, the authorization server responds with a device code, an end-user code and end-user verification Uniform Resource Identifier (URI). Next, the client provides instructions to the end-user to use a user agent on another device and visit the end-user verification URI. After the end-user is authenticated, the authorization server prompts the user to input the end-user code for validation. During this step, when the end-user reviews the client’s request, the device client continually polls the authorization server to identify whether the user has completed the authorization step. Finally, the authorization server validates the device code and issues the access token to the client if the access is granted or if there is an error in case of denial, or it notifies the client to poll the authorization server continually.

Protocols such as Constrained Application Protocol (CoAP) and Message Queuing Telemetry Transport (MQTT) are mainly used for resource-constrained devices, leveraging OAuth 2.0 tokens for authorization purposes [39,40].

- Constrained Application Protocol: CoAP is a protocol specially designed for interaction between endpoints and networks that are resource-constrained [41]. Specifically, this protocol is designed for machine-to-machine applications. The structure of CoAP is logically divided into two layers [42]. The first layer is used for requests and responses. CoAP uses a Representational State Transfer Constraints approach, allowing the clients to use HTTP methods to send requests [42]. The second layer, called the message layer, is used for retransmitting lost packets [42]. CoAP uses the Datagram Transport Layer Security (DTLS) protocol for security.
- Message Queuing Telemetry Transport: MQTT is a messaging protocol for the IoT standardized by the OASIS consortium. MQTT offers bidirectional communications and supports scalability and reliability. MQTT is considered a great communication protocol for the IoT due to its simple, lightweight, and easy deployment properties [43]. Moreover, the use of MQTT has advantages in the ability to work with low-end devices [44], implementing machine learning algorithms in the cloud by interfacing the device with the Internet [45], and easy integration of new devices [45]. MQTT also comes with limitations. The default plain-text data exchange mechanism is a significant threat to data security [43]. Several security attacks on IoT communication protocols were analyzed in [42].

### 3.3. Hybrid User-Managed Access Architecture

UMA is developed as part of the Kantara Initiative [46]. Unlike OAuth, access to third-party applications for resources is granted regardless of where those resources reside. Hence, UMA follows a capability-based approach, in which an entity with a defined capability and an access token will have access to a resource [8,10]. UMA is a user-oriented standard and is evolving to be adopted in IoT environments.

**Figure 4:**
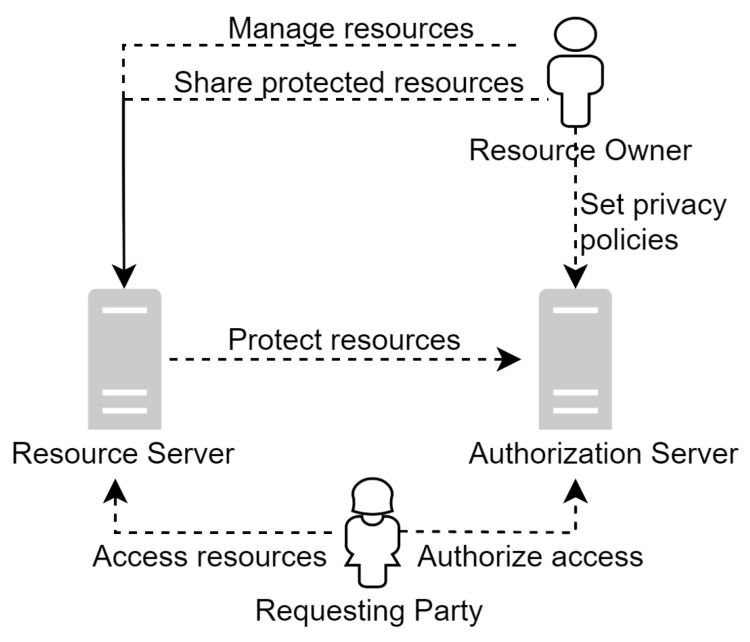
UMA Architecture [10].

Figure 4 shows an example of UMA architecture. A resource owner manages all the resources stored in a resource server. The function of the authorization server is to protect the resource server. The resource server registers the resources that need to be protected with the authorization server and then configures them with appropriate policies for the registered resources. The client first sends a request to the resource server to receive an authorization grant. On the first attempt, the resource server registers the permission with the authorization server and issues a permission ticket to the client. The client presents the ticket to the authorization server. If the permission is granted, the authorization server issues a requesting party token (RPT) to the client. The client uses the RPT to access the requested resources.

### 3.4. Discussion

Among the three authorization architectures, XACML and attribute-based access control in combination can offer rich and fine-grained solutions. The interpretation of attributes and the language used to define the access control policies is complex and makes this standard a limitation in terms of usability [10]. OAuth has several advantages in terms of scalability, interoperability, and flexibility. However, research finds that it lacks a fine-grained property during implementation. Due to the requirement of the user registration, the client registration, and the nature of IoT networks, implementation and configuration are challenging for service providers. UMA provides a unified control point for authorization [47]. There are many use cases where UMA can be utilized for IoT networks. However, the research has identified four major challenges, including availability, transparency, traceability, and maintainability [48]. UMA follows a centralized architecture and therefore is at risk of a single point of failure [48]. In a third-party access control service utilizing UMA, it can be a difficult task to trace the authorization history [48]. In terms of maintenance, it is challenging to upgrade in a centralized solution [48]. The blockchain technology that follows the distributed architecture is being researched to assist UMA for the IoT [48,49].

Many studies have been conducted in adopting the architecture in the IoT [40,48,50]. The fast evolving of IoT and cybersecurity also raises new challenges in these architectures. For example, zero trust architecture is promising in protecting the IoT and enabling data transfer among users, devices, applications and services [51,52]. More studies are desired to study how the architecture supports micro segmentation to realize zero trust in the IoT.

## 4. Access Control Models in IoT

There are many access control models proposed for traditional computing and networking environments. An overview of such models implemented for the IoT and their issues are discussed below.

### 4.1. Access Control Models

1. Discretionary Access Control (DAC): DAC is one of the primary access control techniques introduced in computing. It grants access by managing an access control matrix or an Access-Control List (ACL) [53]. Once access is granted in DAC, it remains forever until the administrator revokes access. In IoT, the access should be continually monitored and evaluated for timely revocation. As new devices are being added or when existing devices are removed, access control must be updated automatically. Access decisions should be made based on various criteria in different situations. DAC is a static model, and the ACL must be manually updated by an administrator. For a dynamic environment such as IoT, DAC is not suitable.
2. Role-Based Access Control (RBAC): In RBAC, a user is granted access based on roles which are in turn assigned with appropriate permissions to access resources [54]. Although it is easy to assign permissions to roles, many users may fall under a single role. As IoT devices come with a variety of functionalities and offer a wide range of services, the administrator must create a new role whenever a device with new functionality is added to a network. In a large enterprise network, this may lead to role explosion. In addition, RBAC may have challenges in supporting dynamicity.
3. Attribute-Based Access Control (ABAC): ABAC is considered by many as one of the suitable models for IoT to provision access rights because of its ability to support additional attributes with user roles. Using ABAC, different attributes of IoT such as device ID and location can be included for evaluation while providing access. Even though this model is being used in large-scale projects such as smart grids, ABAC faces the issue of complexity due to its centralized architecture [17,55].
4. Organization-Based Access Control (OrBAC): OrBAC is an extension of the role-based access control by including a new dimension called “organization” [56]. This additional attribute helps in granting access when multiple organizations play a role or when an organization has many subdivisions. However, other than the above-mentioned concept, this model is no different from its parent model RBAC and is considered unsuitable for heterogeneous and dynamic IoT environments.
5. Usage-Based Access Control (UCON): UCON was introduced as a framework to protect digital resources that come under the digital rights management (DRM). This model comes with three main concepts: authorization, obligation, and condition [11]. The authorization represents evaluation as to whether a subject is eligible to be provided access. The obligation is a criterion that a subject must perform to be provided with or sustain access. The condition represents the criteria that a subject must satisfy. Due to the three evaluation categories, UCON provides high dynamicity where the access is continually monitored, thereby revoking access whenever required by policies. However, this model does not explain the delegation property and follows a centralized architecture.
6. Capability-Based Access Control (CapBAC): The concept of CapBAC was started as part of the IoT@Work project [57]. It is an initiative by the European Union to leverage IoT to automate various services in public sector entities [57]. CapBAC follows a distributed approach. It is implemented through various nodes by using PDP and PEP [58]. In CapBAC, a resource requester must show a particular capability to request an access token. The PDP decides whether to issue the token to the requester. Once issued, the token is evaluated at the PEP for the requester to access the resource. Another advantage of CapBAC is the property of delegation, where nodes can be given the authority to provide access to other nodes. The level of delegation is determined while designing the model. Nevertheless, the model must depend on a central server for either identity verification or certificate to decide whether to trust the requester or not. The access is issued based on the requester’s capability. CapBAC does not consider context while provisioning access [8].
7. Blockchain-Based Access Control (BBAC): Blockchain technology has had explosive growth in security and privacy applications in recent years. The important characteristic of this technology is its distributed nature. The methods through which the blockchain-based access control is described in the literature can be further divided into transaction-based and smart contract-based access control [59,60,61]. Transactions can be used to grant, delegate, or revoke access rights. Smart contracts can evaluate access requests and make decisions based on the access policies defined by the resource owner. In either case, an access token is generated and passed on to the requester, which signifies the right to access. The main disadvantage of the transaction-based approach is that access decisions must be made by a centralized node. In contrast, the smart contract-based approach may invoke large overhead due to the creation of contracts between nodes.
8. Relationship-Based Access Control (ReBAC): Relationships such as user-to-user, user-to-device, and device-to-device relationships can be utilized for identity access management. It is expected by many consumers that the IoT device manufacturers include the concept of relationships for access provisioning. Thus, Identity Relationship Management (IRM) is gaining attention and has been identified as a promising identity and access management (IAM) system for the IoT [62]. In ReBAC, permission is granted based on the relationship between a subject and a device. For example, if a subject is the owner of a device, the device can access a resource. The relationship as an ‘owner’ of the device grants the permission [63]. ReBAC is one of the recent models and it is gaining more attention due to its dynamic nature [64].

In addition to the models discussed above, a few more access control models can also be found in the literature [8,20,65,66]. In the History-Based Access Control (HBAC), an access decision is made dynamically based on the context of access history in a given state. The model requires a centralized authorization system such as a certificate authority in place [65]. Two access control models, Risk Adaptive and Proximity-Based Access Controls [66], are available for implantable medical devices. In the risk adaptive model, a decision is made by considering the risk factor evaluated by policies. In the proximity-based model, a device’s programmer must be in close proximity to a patient to generate the key to decrypt the communications from the device. This model has a potential physical security issue that an adversary should not be near the patient [66]. The proximity-based model is used widely in implantable devices. Trust-based models allow devices to be attached to use spaces within a short period [20]. In this model, the access permissions are assigned to users based on their levels of trust. However, it is difficult to define how trust and relationships are established between users and devices. Examples of trusted-based models include the Billing-Based Access Control and Privilege-Based Access Control [8]. The billing-based approach is a business-driven control where a service is provided to any user who receives an adequate reward [8]. Identity does not matter in this model. In the privilege-based model, a decision is made based on an organization’s policies, and the access is restricted only to particular users [8]. Trust is one of the important criteria in a heterogeneous environment such as IoT. It enhances both security and privacy [67]. However, trust systems in the IoT face challenges such as heterogeneity, scalability and integrity [67].

### 4.2. Discussion

Table 1 summarizes the discussed models including DAC, RBAC, ABAC, ORBAC, CapBAC, UCON, ReBAC and BBAC and their concerns to fulfill the access control requirements. Section 2.2 presents 12 security requirements which are desired in any access control process. The comparison in the Table 1 is limited to the features enabled by the access control models discussed in Section 4.1. As shown in Table 1, most of the access control models discussed do not support the distributed nature of the IoT. This makes the blockchain-based access control model very attractive in the IoT. While all the access control requirements are essential, any access control solution in the IoT must provide the granularity, the interoperability and the scalability, which are appropriate to the associated applications.

The comparison in Table 1 is based on how these access control models support access control requirements in general, and is not based on specific solutions. For example, RBAC has challenges in supporting granularity, complexity, and dynamicity in the IoT. However, RBAC model is also popular in the IoT [68,69] due to its simplicity. ReBAC integrates relationships into access control. However, the existing literature states a significant challenge exists in ReBAC too. The exploding relationships may result in the difficulties of massive relationship management and low compatibility when establishing social relationships between heterogeneous entities [70]. Solutions such as knowledge graphs, and unified gateways could potentially address these challenges, respectively [70]. The BBAC model is promising to fulfill the access control requirements in the IoT. However, challenges have also been found in the BBAC model [71]. The operation of blockchain for access control in IoT is still in its infancy. All access control models have limitations, as shown in Table 1. These limitations indicate that more research efforts are desired in the field.

## 5. Access Control Policies

An access control policy specifies access permissions when an IoT device connects to a network. Access control policies primarily administer and manage the entire access decisions. The process of formulating access control policies for IoT networks should meet several objectives [8]. The process should not be too complex for a device owner to understand, and the usability should be of primary importance to the policy [72]. Further, IoT devices that connect to the network should be flexible to conform to the network’s policies so that risk is not introduced into the network. Due to the nature of the IoT, framing access control policies is domain-specific. Policies must adapt to a particular environment and its characteristics. For example, smart home products available in the market today can facilitate users to generate policies that allow access delegation. However, the generated policies might not be as fine-grained as the users expect and may lead to over-privilege [73,74]. For instance, the Nest thermostat allows a homeowner to add a family member. This will give the family member complete access to the device, although the homeowner might not intend to give the family member full access [73]. Many access control solutions define the properties of delegation and the context which are required for dynamicity. However, this generally happens at a single node when it comes to access decisions or administration. Additionally, various commercial IoT services such as AWS IoT and NiagaraAx support ACL and role-based policies [75]. ACL-based policies are administered manually. It becomes unsuitable for the creation of roles and permissions when devices are added at scale.

### 5.1. Dynamic Policies’ Specification

A comprehensive review of the policies’ specification in the IoT reveals that the existing solutions lack the dynamicity in policy generation, decision and evaluation [76]. Machine learning can be utilized for policies’ automation. With automation, there is no need to edit policies manually when devices are added at scale. Therefore, the use of machine learning will directly help in achieving dynamicity in an access control solution. Specifying access control policies at runtime is desired to fulfill many requirements, as discussed in Section 2.2. This section summarizes the techniques adopted for dynamic policies specification in the IoT.

Traditional Access Control Model-Based Approaches: Liu et al. proposed an access control model for resource sharing in [77]. The approach is based on RBAC. The authorization mechanism uses a planning graph-based technique to search for an optimal authorization route for administrators to grant privileges for a subject. The policy encompasses user roles, permissions and resources. This solution faces challenges in terms of resource efficiency and scalability. In [34], Alkhresheh et al. designed a dynamic access control framework based on ABAC. A novel algorithm, namely the automatic policies specification algorithm, in which the policy is generated based on the extraction of the attributes from the subject, the object and the operation to be performed, and is evaluated against a set of primitive facts, was presented. In addition, the policy enforcement algorithm adjusts the policies continually and automatically. In [78], Gabillon et al. proposed an ABAC-based framework for the MQTT protocol to which sensors could subscribe for topics. In their approach, the policy language is based on the Shapes Constraints Language introduced by the World Wide Web Consortium. Although the policies are expressive and contextual by means of attributes, the administration is still static. In [79], Riad et al. extended XACML from adaptive policies to suit the distributed IoT environment. Their architecture follows the ABAC model and allows the policies to be adjusted at runtime [79]. The generated policies are validated using the MD5 message-digest algorithm checksum. The scheme protects the IoT network from two attacks, i.e., the masquerade attack and the man-in-the-middle attack. Similarly, a conceptual framework that enforces access control policies in a smart health environment was proposed in [80]. This framework follows a centralized architecture but can refine policies at runtime to ensure dynamicity. The policy language is based on XML. XML was utilized due to its flexibility to exchange policies between domains. The framework in [80] is based on the ABAC model. In fact, many approaches utilize the ABAC model to enforce access control policies due to its support for multiple attributes. However, the ABAC model may also have performance issues compared to others due to multiple attributes used for access control [81].

Artificial Intelligence-Based Approaches: Bertino et al. conducted a case study on XACML policies to analyze their model developed based on symbolic learning in the Generative Policy Model (GPM) in [36]. A public dataset, including XACML policies’ requests and responses, was used to perform the study. Based on the dataset, they generated a set of examples that contain ABAC parameters that were based on answer set grammar (ASG). Cunnington et al. proposed a centralized architecture based on GPM for connected and autonomous vehicles in [33]. The adopted method is based on inductive logic programming. Their solution does not generate access control policies, but it refines and stores policies dynamically. Liu et al. proposed a risk prediction-based access control model for the Internet of Vehicles (IoV) in [82]. In their approach, they use a centralized architecture with a generative adversarial network (GAN) model based on Long Short-Term Memory (LSTM) to improve the training dataset. The vehicle can access the requested resource if the risk is below a predefined threshold. Yu et al. proposed a learning-based approach that learns contextual access control policies from the behavior patterns of multiple smart home devices in [83]. This approach uses a federated learning framework that incorporates temporal modeling. In [84], Chu et al. proposed a multi-access control technique based on battery prediction with energy harvesting in IoT. The proposed solution utilizes a LSTM-based deep neural network. It is designed for a wireless network where sensor nodes are dispersed geographically. The nodes are granted access to the base station based on the sensor node’s battery state. In the proposed two-layer LSTM network, the first layer predicts and generates the battery level of the sensor node. The second layer uses the channel information and predicted values to generate access control policies.

Blockchain-Based Access Control Approaches: Blockchain has been explored to make access control decisions in IoT due to its distributed nature. A smart contract-based access control solution was proposed in [85]. In the blockchain-based approach, a policy created by a resource owner is stored in the blockchain as a transaction. The policy is written in XACML and is transformed into a smart contract. To update or delete a policy, the contract is replaced with a new smart contract. In [86], Liu et al. proposed a distributed ledger-based approach to protect the privacy of IoT data. In the approach, policy updates are conducted through the edge node by adding a new policy to the blockchain, thus enabling the dynamic access control. In [87], a distributed blockchain-based access control solution is proposed for the smart grid domain. The approach consists of three layers: the first layer is the network layer, the second layer consists of the raw RBAC and ABAC policies, and the third layer consists of the distributed ledger. Context information updates to the PDP are performed by virtual auditors. These updates assist the PDP in performing dynamic access control decision making. In [88], Zhang et al. proposed a smart contract-based access control approach utilizing the ABAC paradigm. The policies are not hard coded in the smart contracts, allowing the approach to have less overhead. This solution also contains predefined functions to add, delete and update the policies, thus assisting the concept of the dynamic access control.

Policies that Carry Data: With the contextual nature and the amount of sensitive data transmitted and processed, IoT devices can also embed policies within the data. This embedded data policy allows for constant monitoring and revocation of access. First introduced in [89], sticky policies provide a data owner-centric approach for the IoT and allow users to embed policies into data. This concept was applied in many approaches in the field of IoT. For example, an approach called the policy-carrying data was proposed in [90]. In the approach, the policy can specify information regarding permissions, obligations, and restrictions of the data, which brings dynamicity. The policy language is based on first-order logic. However, the language is considered complex, and there is a need for a centralized server to evaluate both data producers and consumers. In [91], Sicari et al. use a middleware architecture to handle policy requests and responses by utilizing ABAC. The approaches in [37,92] use sticky policies by utilizing the edge computing architecture. JavaScript Object Notation format was used to define policies. End-to-end communication was encrypted to preserve data privacy. Sticky policies allow for intelligent control over the authorization of IoT resources. However, it comes with limitations too. There is no established language for policies due to the pinning of the policies with the data. It may also increase the computational overhead on the devices due to the encryption that is being used during data transmission [93].

### 5.2. Discussion

The challenges faced by the current dynamic policies’ specification solutions are discussed below. Table 2 summarizes the contributions and the challenges in the reviewed solutions.

Centralized Architecture vs. Distributed Architecture: A number of solutions including [32,33,36,90] have adopted centralized architecture to specify dynamic policies. For a heterogeneous environment, centralized architecture is complex to design, and does not scale well. Even though many IoT networks depend on cloud platforms for management, it is recommended to utilize technologies such as edge computing, which supports distributed architecture. The solution proposed in [83] utilizes an edge computing paradigm [94] to learn context-aware access control policies from multiple smart homes.

Policy Generation, Decision and Evaluation at Runtime: Many solutions in the literature proposed their approaches for dynamic policies’ specification. For example, the solution in [32] uses supervised machine learning to classify the device access behavior based on a real-life dataset. Access control solutions should be able to make access decisions based on policies independently. Many scenarios may occur, such as the failing connection to the central policy management server [33], a large-scale project such as a smart city where numerous devices are added at scale [95], and any unforeseen context where a policy may not exist. An access control solution should consider these scenarios and design capabilities to generate and evaluate policies at runtime.

Eliminating Policy Violation and Policy Conflict: IoT devices are often used to automate physical processes such as detecting water leaks, adjusting temperature, controlling security cameras, and enabling autonomous driving. Hence, dynamic policies’ specification or policies’ automation should address policy conflict and policy violation identified from the generated policies. Policy validation provides the opportunity to resolve several issues related to the security of the devices and physical safety.

Selection of Required Features: A number of solutions such as [82,83,84] utilize machine learning approaches for extraction or refining policies at runtime. The machine learning-based solutions depend on a specific set of defined features for operations. The features used in machine learning in IoT include, but are not limited to, the contextual attributes of the subject that requests access, resources and other environmental attributes. When implemented in real time, frequent requests to the current state or attribute values may potentially reduce the performance of the devices. Therefore, the machine learning solutions must consider the memory and processing capabilities while performing feature selection.

Accuracy of Real-Time Classification: Access control authorizes a user or a device’s request to access a particular resource. Hence, in a classification scenario, a machine learning solution must predict a request with the utmost accuracy. Otherwise, at times of misclassification, there is a chance that a legitimate subject might be denied access. In generative models, it is believed that any policy that is being generated should not conflict with the existing policies in the repository and should not violate the security and privacy requirements of the network. Designing a solution to verify these issues automatically is a challenge.

Lack of Public Balanced Datasets for Research: Unfortunately, identifying a relevant publicly available dataset is a challenge for access control research in IoT. Various constraints such as security and privacy might be part of the reasons. However, the machine learning models should learn from a balanced dataset to provide accurate classification or policy generation. For example, Bertino et al. evaluated their proposed solution with the help of a noisy XACML dataset [36]. Their models led to issues such as overfitting. The work in [82] utilized dataset from an intrusion detection project. A well-balanced dataset is essential to propose novel machine learning-based access control solutions effectively.

Real-Time Implementation: A number of the proposed solutions have not been evaluated in real time. For example, studies such as [35,37,78,80,91,96,97] have been proposed as generalized frameworks that can be utilized for dynamic policies’ specification in the IoT. Solutions tend to behave differently in a test environment and a real-time environment. Consequently, when they are implemented in real time, the actual issues and the challenges the solutions may face shall be captured, enhancing the scope for further research.

## 6. Access Control Research Challenges and Future Directions

This section summarizes the research challenges in the access control and points out future research directions in the IoT.

### 6.1. Research Challenges

Table 1 and Table 2 reveal the gaps in access control in the IoT. Many challenges exist in designing an access control solution to fulfill the access control requirements identified in Section 2.2.

Identities of Things: The access control assumes IoT devices can be uniquely identified and access control policies can be applied to network traffic. As users are identified in a digital network by their unique identities, IoT devices also require their unique identities when connecting to a network. Identities of Things (IDoT), a general term describing IoT entities (e.g., users and devices), has been adopted. IDoT is a research area that has progressed towards modeling identities of physical entities. IDoT includes identities of both users and devices. Identities of users have been studied extensively. Four primary authentication factors could be used to identify users: something you know (e.g., username and password), something you possess (e.g., a physical token or a smart card), something you are (e.g., fingerprint or face recognition) and something you do (e.g., voice or sign). IoT devices can only be identified by something they have. A common technique to identify a device in a network is using the device’s MAC address. However, the MAC address can be easily spoofed.

In general, an identity in the IoT consists of a set of attributes and dynamic values along with the member in varying contexts [98]. It can be a collection of things, should have a purpose, and should be treated uniformly across platforms. There are many representations of identities. Identities can be based on globally unique identifiers [99,100], a combination of user characteristics [101], a set of attributes of the users [102] or a set of claims [103]. These approaches all possess a commonality based on the fact that they link an identity uniquely to a particular entity [98]. Furthermore, the work in [98] suggests that unique identities are not suitable in terms of policies’ specification and policy management. On the other hand, behavioral fingerprinting shall become efficient as they possess dynamic characteristics. Due to the enormous amount of devices available, the scalability of new schemes is essential. Most of the current strategies for identification are based on symmetric or asymmetric cryptography. However, both the cryptographic techniques have limitations when they are used for identification [104]. Given the heterogeneity and the need to protect the data that IoT devices collect, IDoT needs to be addressed before access control [105].

Heterogeneity, Resource Constraints and Interoperability Issues: Any access control solution should be designed to address the complex nature of the IoT. IoT networks are heterogeneous networks. They often include multiple administrative domains due to the scale of the networks. As data migrates from one domain to another, there are also concerns about the security and privacy of the data, since it is hard to trace and manage the data ownership without a central trusted authority [106]. Due to its distributed nature and property of delegation, the blockchain is well suited to IoT networks. However, it is still challenging for the blockchain-based access control to fulfill all the requirements at the same time. Storing a large volume of data in a blockchain proves to be costly. Security is a major concern while integrating a blockchain with an off-chain data store [71]. The performance of the solutions appears to be another major concern [107]. Furthermore, the storage and processing of private data in enterprises is a barrier to utilizing complete decentralized architecture [71]. More research on the blockchain is needed for access provisioning in IoT environments.

IoT devices also come with a number of constraints, particularly in terms of memory, processing power and battery. The sensors deployed in harsh conditions and used to check abnormality rely on an external energy source [108]. Hence, the constant transmission of data or transmission of large payloads will invoke overhead and drain battery power significantly. The proposed solutions should not invoke any such overhead on the devices and reduce their performance. One way to overcome this challenge is to bring the computation and storage to the edge, which may enhance the network’s performance [109].

Not all the devices come from the same manufacturer in an IoT network. Hence, the data that represents the devices may vary in formats. It is challenging to extract and integrate such forms of data and achieve uniform information. A number of protocols utilized for device-to-device communications are not interoperable in nature [110]. When connected to a network, devices should be interoperable, allowing access control to function as expected. However, achieving syntactic, semantic, and cross-domain interoperability in IoT is a challenge [108].

Access Control Security: The security of the access control process itself is also a concern. Access control is one of the most important services in security. Therefore, the access control solution itself should be resistant to attacks. The security flaws in the access control may occur in many places, including design, protocols, implementations and configurations. Although many access control models have been proposed for IoT, limited research has been conducted on access control process security analysis [16]. Due to the importance of access control for any network, access control security analysis is desired. Moreover, IoT networks may collect a large amount of sensitive information. Thus, the access control policies must comply with privacy regulations such as the General Data Protection Regulation (GDPR) and Health Insurance Portability and Accountability Act (HIPAA).

### 6.2. Future Directions

Access control is essential to secure the IoT. Future research is desired in the following areas:

Developing New Scalable Schemes for Identities of Things: Identities of Things comprises the identities of both humans and devices. Identifications of humans have been studied extensively. Research in the area of device identification is still emerging. Due to the enormous amount of devices available, the scalability of new schemes is essential. Most of the current strategies for identification are based on symmetric or asymmetric cryptography. However, both the cryptographic techniques have limitations when they are used for identification [104]. New schemes for identification should be further studied, including decentralization using blockchain, modeling the identity of devices using their behavioral patterns and device fingerprinting.

Enabling Novel Multi-factor Authentication (MFA) Methods for the IoT: The MFA method provides various approaches to verify a user’s identity. MFA is effective for internet-based applications and services. As most IoT-based services are dependent on the Internet, MFA is essential and desired for IoT-based applications. However, many IoT devices do not come with screens and keyboards. Hence, implementing MFA in IoT applications is challenging. Further research should consider proposing novel MFA methods for devices with small form factors.

Utilizing Relationships for Authentication and Access Control: The definition and utilization of relationships among users, devices, applications, and other services can provide dynamic intelligence, which can further be used for authentication and access control. Challenges exist in the definition and characterization of relationships, establishing relationships among heterogeneous entities, using relationships for authentication and access control, and managing relationship explosion. Hence, future research in this area should focus on proposing novel solutions to address these challenges.

Resolving Interoperability Issues by Standardization: With the growing number of devices and their applications adopted by communities, there is a need for a borderless IAM system. The system needs to be built in a modular and pluggable manner without requiring a single organization to maintain them. Global standardization bodies should define specifications for such systems, which will help resolve the challenges when multiple information systems are adopted in an organization. Using a common language to extract the attributes and behavior of entities, as well as the machine learning-assisted automated policy generation, generic APIs are some future initiatives to enhance interoperability.

Adopting Zero Trust Architecture for the IoT: The adoption of zero trust architecture in IoT is an emerging research area. Zero trust is a comprehensive approach to access provisioning across a network that is primarily based on network segmentation. It suggests continuous monitoring, verification, and adjustment of policies. IoT networks can potentially extend beyond the perimeter of an organization. Although the literature on zero trust architecture for IoT exists, there is still scope for more research based on zero trust security architecture. Future research in this area may focus on modeling dynamic access control solutions, the impact of zero trust on the interactions between different entities and the adoption of zero trust in large-scale networks.

## 7. Conclusions

This paper conducts a comprehensive survey on access control in the IoT. Access control is an essential security service in the IoT. Due to the variety of the IoT devices, the resource constraints on the IoT devices, and the heterogeneous nature of the IoT, the design and implementation of access control in the IoT are challenging. The paper identifies and summarizes twelve requirements for access control in the IoT. These requirements include providing the desired granularity; supporting policies’ specification for dynamicity and delegation; handling the complex nature of the IoT networks; supporting interoperability among different manufactures; facilitating easy access management for users; automating the processes of policy generation, decision, and evaluation; overcoming resource constraints on IoT devices; ensuring coherence across multiple administrative domains; resolving device identification issues; minimizing the downtime; being scalable; being resistant to any cyber attack.

The access control grants or denies requests from a user, a device, an application or a service. An access control process includes essential functions such as the authentication function, access control function and managing policies’ function as shown in Figure 1. Three common types of authorization architectures can be found in the IoT, i.e., the policy-based architecture, the token based OAuth architecture and the hybrid UMA architecture. The policy-based architecture can offer rich and fine-grained solutions for the IoT. It faces challenges in terms of usability due to the complexity of the IoT. The token base OAuth architecture has advantages in scalability, interoperability and flexibility. However, it lacks a fine-grained property during implementation. The hybrid UMA architecture provides a unified control point for authorization. However, it faces challenges including availability, transparency, traceability and mutability. The integration of these architectures with zero trust needs to be further studied.

Many access control models have been adopted in the IoT. This paper provides an overview of eight access control models. These access control models include the discretionary access control, role-based access control, attribute-based access control, organization-based access control, usage-based access control, capability-based access control, blockchain-based access control and relationship-based access control. The paper further evaluates and compares the existing access control models fulfilling the access control requirements as shown in Table 1. The comparisons demonstrate that none of the existing access control models meet all the desired requirements. The gaps between the existing access control models and the access control requirements indicate that more research efforts are desired in the access control in the IoT.

Access control policies developed for access control models must handle the dynamic nature of the IoT. Solutions proposed for the traditional access control models are static and based on centralized architecture. Many machine learning-based approaches are proposed for dynamic policies’ specification in the IoT. Machine learning-based approaches are great at handling the dynamic nature of the IoT and meet the access control requirements such as scalability and automation. However, machine learning-based approaches also face challenges including selecting appropriate features for machine learning, the need of a large balanced dataset, the requirement for high accuracy of the classification and meeting the real-time requirement. The centralized architecture may have challenges in design and scalability in a heterogeneous environment such as the IoT. Blockchain-based access control approaches have also been explored to make access control decisions in the IoT due to its distributed nature.

Many challenges exist in designing access control solutions for the IoT. These challenges include addressing identities of things issues in the IoT, utilizing relationships for access control, supporting policies’ specification and automation, resolving interoperability issues, integrating blockchain with access control, overcoming resource constraints on IoT devices and ensuring security of the access control process in the IoT. The development of new scalable schemes for identities of things, enabling novel multi-factor authentication methods for security, utilization of relationships for authentication and access control, and resolving interoperability issues by standardization are desired to fulfill access control requirements in the IoT.

Due to the variety of IoT applications, there is no one-size-fits-all solution for access control in the IoT. The access control requirements are often intertwined with one another. Therefore, it is possible that satisfying one requirement automatically paves the way to fulfilling another. IoT raises many security and privacy issues due to the amount of data collected. Despite the challenges encountered in designing and implementing access control in the IoT, it is desired to have an access control solution to meet all the identified requirements to secure the data in the IoT.

## Figures and Tables

**Table 1:** Access Control Models and Requirements.

Features	DAC	RBAC	ABAC	OrBAC	CapBAC	UCON	ReBAC	BBAC
Granularity	Coarse	Coarse	Fine	Coarse	Coarse	Fine	Fine	Fine
Context-Aware	No	No	Yes	Yes	No	Yes	Yes	Yes
Dynamicity	No	No	Yes	No	Yes	Yes	Yes	Yes
Distributed Nature	No	No	No	No	Yes	No	No	Yes
Interoperability	No	No	Yes	Yes	Yes	No	Yes	Yes
Delegation	No	No	No	No	Yes	No	Yes	Yes
Revocation	No	No	No	No	Yes	Yes	Yes	Yes
Scalability	No	No	Yes	No	Yes	Yes	Yes	Yes

**Table 2:** Analysis of Dynamic Policies’ Specification Approaches.

	Contributions	Challenges
[77]	RBAC-based approach for resource sharing	Scalability and resource efficiency
[34]	Extended XACML and added three	No verification of who is going to use
	functionalities to implement adaptive policies	the access decisions
[78]	An ABAC-based framework for the MQTT	The approach is not tested in real time
	protocol	
[79]	An adaptive XACML policy-based approach	Utilization of many attributes may
	to specify access control decisions	potentially affect the performance
[80]	An attribute-based access control	The solution is a generic framework. It
	framework for smart health applications	is not implemented in real time
[36]	ASG-based architecture to generate	Noisy dataset which may result in
	policies at runtime	conflict policies
[33]	Proposed an architecture to generate runtime	Centralized architecture
	policies for autonomous vehicles	
[82]	Risk-based access control approach	Computation time is high
	based on LSTM and GANs	
[83]	Federated learning approach to learn	Real-time implementation
	policies at runtime	
[84]	Distributed technique for battery state	Efficiency depends on energy
	prediction for remote sensor devices	availability in sensors
[85]	ABAC policies are coded in smart contracts	Huge storage space requirement,
	and executed as distributed smart contracts.	computational overhead
	Utilizes Ethereum protocol	
[86]	Distributed and dynamic access control based	Susceptible to tampering
	on blockchain and fog computing	
[87]	A three-layer interconnection architecture	Interoperability
	to enforce policies for smart grid	
[88]	Smart contract-based framework and	Throughput
	ABAC model for access decision-making	
	in smart cities	
[90]	A formal model using first-order logic	Complex language, centralized
	to regulate data access, and a computational	architecture
	model to verify policies	
[91]	Dynamic policy enforcement framework	Real-time implementation
	with a distribution and synchronization	
	system	
[92]	Decentralized privacy enforcement	Information flows to be declared
	framework using sticky policies	beforehand
[37]	Middleware architecture to distribute	Testing performed in simulation.
	and update policies in an IoT environment	The exact implications need to be
		tested in real time

## Data Availability

Not applicable.

## Abbreviations

The following abbreviations are used in this manuscript:

ABAC	Attribute-Based Access Control
ACL	Access-Control List
ASG	Answer Set Grammar
BBAC	Blockchain-Based Access Control
CapBAC	Capability-Based Access Control
CoAP	Constrained Application Protocol
DAC	Discretionary Access Control
DoS	Denial of Service
DRM	Digital Rights Management
DTLS	Datagram Transport Layer Security
GAN	Generative Adversarial Network
GDPR	General Data Protection Regulation
GPM	Generative Policy Model
HBAC	History-Based Access Control
HIPAA	Health Insurance Portability and Accountability Act
HBAC	History-Based Access Control
IAB	Internet Architecture Board
IDoT	Identities of Things
IETF	Internet Engineering Task Force
IAM	Identity and Access Management
IoT	Internet of Things
IoV	Internet of Vehicles
IRM	Identity Relationship Management
LSTM	Long Short-Term Memory
MFA	Multi-Factor Authentication
MQTT	Message Queuing Telemetry Transport
OAuth	Open Authorization
OrBAC	Organization-Based Access Control
PAP	Policy Administration Point
PDP	Policy Decision Point
PEP	Policy Enforcement Point
PIP	Policy Information Point
PRP	Policy Refinement Point
RBAC	Role-Based Access Control
ReBAC	Relationship-Based Access Control
RPT	Requesting Party Token
UCON	Usage-Based Access Control
UMA	User-Managed Access
URI	Uniform Resource Identifier
XACML	Extensible Access Control Markup Language
XML	Extensible Markup Language
